# Mycophenolate mofetil for corticosteroid-resistant immune-related cystitis associated with tislelizumab: a case report

**DOI:** 10.3389/fimmu.2026.1802747

**Published:** 2026-06-03

**Authors:** Lingping Kong, Fanlu Meng, Jing Yang, Kunlong Tang, Jian Xu, Diansheng Zhong, Linlin Zhang

**Affiliations:** 1 Department of Medical Oncology, Tianjin Medical University General Hospital, Tianjin, China; 2 Department of Pathology, Tianjin Medical University General Hospital, Tianjin, China; 3 Departmentt of Urology, Tianjin Medical University General Hospital, Tianjin, China; 4 Gene Amplification Laboratory, Tianjin Institute of Hepatology, Tianjin Second People’s Hospital, Tianjin, China

**Keywords:** corticosteroid refractoriness, cystitis, immune checkpoint inhibitors, immune-related adverse events, mycophenolate mofetil, tislelizumab

## Abstract

Immune-related cystitis is an uncommon immune-related adverse event (irAE) linked to immune checkpoint inhibitors (ICIs), and managing cases refractory to corticosteroids remains clinically challenging due to the lack of standardized treatment guidelines. We describe a 56-year-old male with extensive-stage small-cell lung cancer who developed corticosteroid-refractory immune-related cystitis following fourth-line therapy with Tislelizumab combined with irinotecan and anlotinib. The patient presented with frequent urination, urgent urination, and dysuria. Urine cultures were negative, and antibiotic treatment failed to alleviate symptoms, leading to a diagnosis of immune-related cystitis (CTCAE grade 3). Initial treatment with methylprednisolone 80 mg/day provided symptom relief, but symptoms recurred when the dose was tapered. Even after escalating the dose to 40 mg/day, symptoms persisted, confirming corticosteroid refractoriness. Combination therapy with prednisone 30 mg/day and mycophenolate mofetil (MMF) 1 g once daily resulted in symptom resolution within 1 week, with normalization of urine routine findings by 8 weeks. Corticosteroids were successfully tapered off without recurrence. This is the first reported case of successful MMF use for ICI-related cystitis, offering a new therapeutic option for corticosteroid-refractory cases. We also discuss considerations for ICI reinitiation to provide a clinical reference.

## Introduction

Immune checkpoint inhibitors (ICIs) have become a core treatment for advanced malignancies by blocking the PD-1/PD-L1 pathway to activate anti-tumor immunity (1). However, these agents can also disrupt immune tolerance, leading to systemic immune-related adverse events (irAEs) affecting multiple organs. Urinary system irAEs are relatively rare, and immune-related cystitis is often misdiagnosed due to its similarity in symptoms to infectious cystitis (2, 3).

A more challenging clinical arises when irAEs fail to respond to standard-dose corticosteroids or become refractory to such treatment, as there is no consensus on optimal management (4). Currently reported cases of corticosteroid-refractory ICI-related cystitis mostly rely on TNF-α inhibitors such as infliximab for treatment (5). Mycophenolate mofetil (MMF) as a well-established immunosuppressant, has demonstrated efficacy in treating ICI-related hepatitis and nephritis (6), but its use in immune-related cystitis has not been previously documented, despite multiple case reports detailing the clinical spectrum of this irAE (7).

Additionally, deciding whether to reinitiate ICIs after controlling irAEs is a common clinical dilemma—especially for tumor types with limited treatment options, such as small-cell lung cancer—requiring a thorough balance of potential benefits and risks. Herein, we report the first case of successful treatment of Tislelizumab-related corticosteroid-refractory immune-related cystitis with MMF, detailing the full multi-line treatment course and discussing the rationale and considerations for ICI reinitiation. This case aims to provide new insights for managing this rare adverse event and guiding decisions regarding ICI reinitiation.

## Case presentation

### General information

A 56-year-old male presented to the hospital in August 2023 with a 2-week history of cough and sputum production. Past medical history included multiple colonic polyps and atrophic gastritis; he had a 30-year smoking history (20 cigarettes per day) and denied alcohol consumption. He had no history of chronic diseases such as hypertension, coronary heart disease, or diabetes, and no history of drug allergies.

### Diagnosis of primary disease and multi-line treatment course

#### Diagnosis

On August 25, 2023, chest CT showed a soft tissue density mass in the right hilar region. On September 5, 2023, pathological examination of bronchoscopic biopsy specimens confirmed small-cell neuroendocrine carcinoma. The patient was diagnosed with right lung small-cell lung cancer (cT4N3M1, extensive stage).

First-line Treatment (September 12, 2023–June 6, 2024):

The patient received durvalumab combined with etoposide and cisplatin for 4 cycles, followed by durvalumab monotherapy for maintenance (cycles 5–10). Efficacy evaluation: Partial response (PR) after cycle 2; progressive disease (PD) was noted after cycle 10 (June 21, 2024). Progression-free survival (PFS) was 9 months, with a platinum-free interval of 7 months.

Second-line Treatment (June 29, 2024–July 27, 2024)

Treatment consisted of durvalumab plus etoposide and cisplatin for 2 cycles. Efficacy evaluation: Abdominal PD after cycle 2. PFS was 1.5 months.

Third-line Treatment (August 31, 2024–October 25, 2024):

Tarlatamab for 3 cycles. Efficacy evaluation: Abdominal PD after cycle 3. PFS was 2 months.

Fourth-line Treatment (November 27, 2024–September 20, 2025)

Irinotecan, Tislelizumab, anlotinib (cycles 1–3); anlotinib monotherapy (cycle 4); irinotecan, anlotinib (cycles 5–7), followed by anlotinib maintenance. Efficacy evaluation: PR after cycle 2; hepatic PD on September 24, 2025. PFS was 10 months (Tislelizumab was discontinued due to immune-related cystitis during treatment).

Fifth-line Treatment (September 30, 2025–October 21, 2025)

A follow-up examination on September 24, 2025, revealed new hepatic metastases. The patient received intravenous lurbinectedin combined with hepatic interventional therapy for 2 cycles. Efficacy evaluation: Stable disease (SD) of chest lesions, but new brain metastasis (small nodule in the right frontal lobe) was identified on November 1, 2025. PFS was 1.5 months.

Sixth-line Treatment (November 10, 2025–December 2025)

The patient received oral temozolomide, Olaparib, and hepatic interventional therapy. PFS was 2months.

As of December 2025, the patient was initiated on palliative symptomatic treatment due to disease progression and poor performance status.

### Clinical course, diagnosis, and treatment of immune-related cystitis

#### Onset of symptoms and initial evaluation

In February 2025, following the 3rd cycle of fourth-line treatment (Tislelizumab, irinotecan, anlotinib), the patient developed recurrent urinary irritation symptoms, including frequent urination, urgent urination, dysuria, and a persistent feeling of incomplete bladder emptying. Given the clinical suspicion of infectious cystitis, he was treated with oral levofloxacin for 14 days, but no improvement was observed.

Initial Auxiliary Examinations

Routine urine test (February 22, 2025): Urinary albumin 3+, urinary leukocyte esterase 3+, red blood cells 116/HPF, white blood cells 453/HPF, indicating significant urinary inflammation.

Routine blood test (February 22, 2025):White blood cells 4.56×10^9^/L, neutrophils 2.77×10^9^/L, platelets 221×10^9^/L, hemoglobin 129 g/L, with no obvious systemic inflammatory changes.

Renal function: Before cystitis onset (January 2025): Serum creatinine (Scr)= 78 μmol/L, eGFR = 92 mL/min/1.73m² (normal renal function). During cystitis (February–May 2025): Scr ranged from 76 to 82 μmol/L, and eGFR from 88 to 94 mL/min/1.73m², with no upward trend despite active bladder inflammation.

Urine culture: Three consecutive urine cultures were negative, ruling out bacterial infection.

Imaging examinations: Abdominal CT (February 24, 2025): Thickened and rough bladder wall, increased density in the surrounding fat space, suggesting bladder mucosal inflammation.

Preliminary Diagnosis and Initial Glucocorticoid Treatment:Based on the patient’s history of Tislelizumab use, typical urinary irritation symptoms, negative urine culture, imaging evidence of bladder inflammation, and exclusion of infection, tumor metastasis, and chemotherapy-related injury, a preliminary diagnosis of Tislelizumab-related immune-related cystitis (CTCAE grade 3) was made.

In late February 2025, the patient received intravenous methylprednisolone 80 mg/day for 2 days, and urinary symptoms significantly relieved.

On March 7, 2025, Tislelizumab was discontinued to avoid further immune-mediated injury; the antitumor regimen was adjusted to oral anlotinib 12 mg on days 1–14 (every 3 weeks) plus oral prednisone 30 mg/day for maintenance. During this period, urinary symptoms remained well-controlled.

#### Difficulty in glucocorticoid tapering and symptom recurrence

On March 31, 2025 (5th cycle of fourth-line treatment), irinotecan + anlotinib was resumed to optimize antitumor efficacy. Prednisone was gradually tapered to 15 mg/day, and urinary irritation symptoms (frequent urination, urgent urination, dysuria) recurred shortly afterward.

The prednisone dose was increased back to 30 mg/day, but symptoms failed to improve. On April 16, 2025, the patient received intravenous methylprednisolone 40 mg/day for 7 days, leading to temporary symptom relief; however, symptoms recurred again after discharge while maintaining oral prednisone 30 mg/day. This pattern of recurrence with tapering and persistent symptoms despite dose escalation confirmed glucocorticoid-refractory immune-related cystitis.

#### Definitive diagnosis via endoscopic and pathological examinations

**Figure 1:**
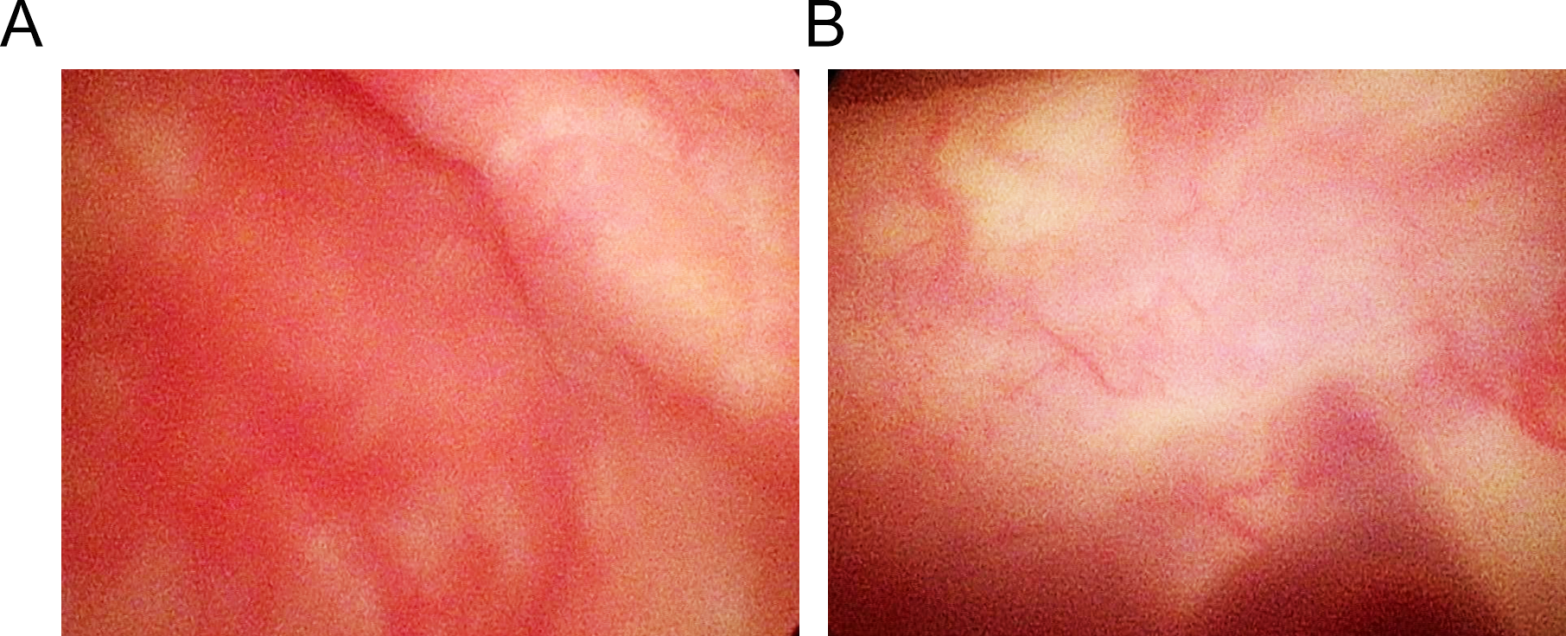
Cystoscopy at the initial examination showed diffuse mucosal redness **(A, B)**.

**Figure 2:**
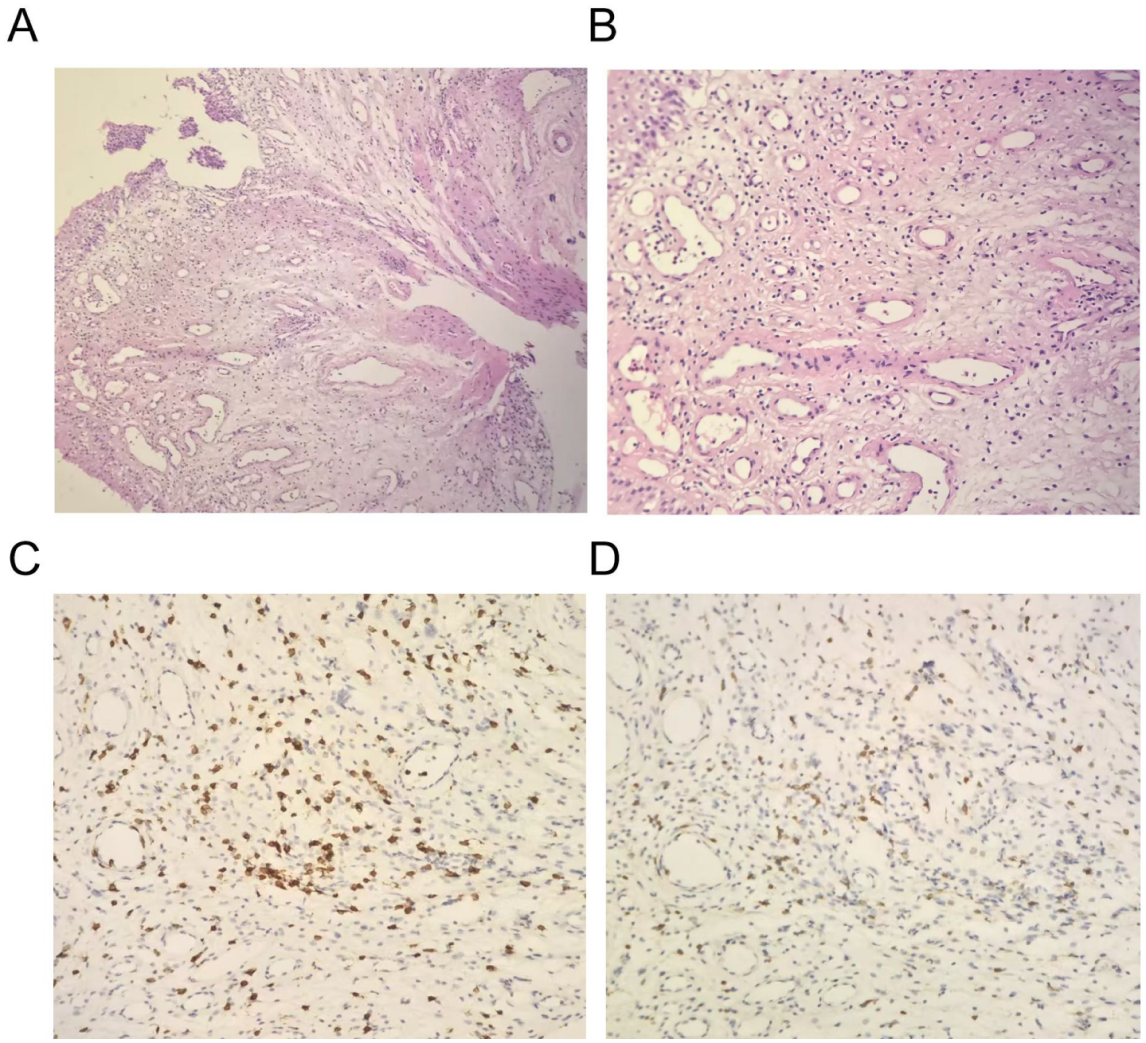
**(A, B)** Bladder biopsy revealed cystitis with lymphocytic infiltration.s (H&E staining, **(A)** magnification×40, **(B)** magnification×100). **(C, D)** Immunohistochemical staining showed a higher number of CD8+ cells than CD4+ cells (magnification×100).

To confirm the diagnosis and rule out other etiologies, further invasive evaluations were performed: Cystoscopy (May 6, 2025):Diffuse hyperemia and edema of the bladder mucosa (**Figure 1**), consistent with inflammatory changes, with no signs of tumor infiltration or ulceration. Bladder biopsy and immunohistochemistry (**Figure 2**): Pathological examination showed chronic mucosal inflammation, granulation tissue proliferation in the stroma, urothelial hyperplasia, and scattered lymphocyte infiltration in the lamina propria. Immunohistochemistry revealed a predominance of CD3^+^ T lymphocytes, significantly more CD8^+^ cells than CD4^+^ cells, and a small number of CD20^+^ B lymphocytes. These findings provided definitive evidence of immune-mediated bladder injury, confirming the diagnosis of Tislelizumab-related immune-related cystitis (CTCAE gade 3, glucocorticoid-refractory).

#### Combined treatment with glucocorticoids and MMF

After a multidisciplinary consultation on May 29, 2025 (7th cycle of fourth-line treatment), the treatment regimen was adjusted to oral prednisone 30 mg/day combined with oral MMF1 g once daily (targeting refractory immune-mediated inflammation).

Throughout the entire clinical course (onset, treatment, and follow-up) of immune-related cystitis, no immune-related adverse events involving other organ systems (e.g., thyroid, muscular, gastrointestinal, dermatologic) were identified.

### Treatment outcomes

Within 1 week of combined treatment, Urinary frequency and urgency significantly relieved, and dysuria improved.

After 4 weeks, routine urine test showed white blood cells 102/HPF, red blood cells 1/HPF, with marked reduction in inflammatory indicators.

After 8 weeks (August 18, 2025), routine urine test returned to normal (urinary albumin -, leukocyte esterase -, red blood cells 0/HPF, white blood cells 0/HPF), and all urinary symptoms resolved.

Adverse reaction: Mild diarrhea occurred during MMFtreatment, which resolved spontaneously after discontinuing the drug without additional intervention.

Subsequent Dose Reduction and Long-Term Follow-Up

**Figure 3:**
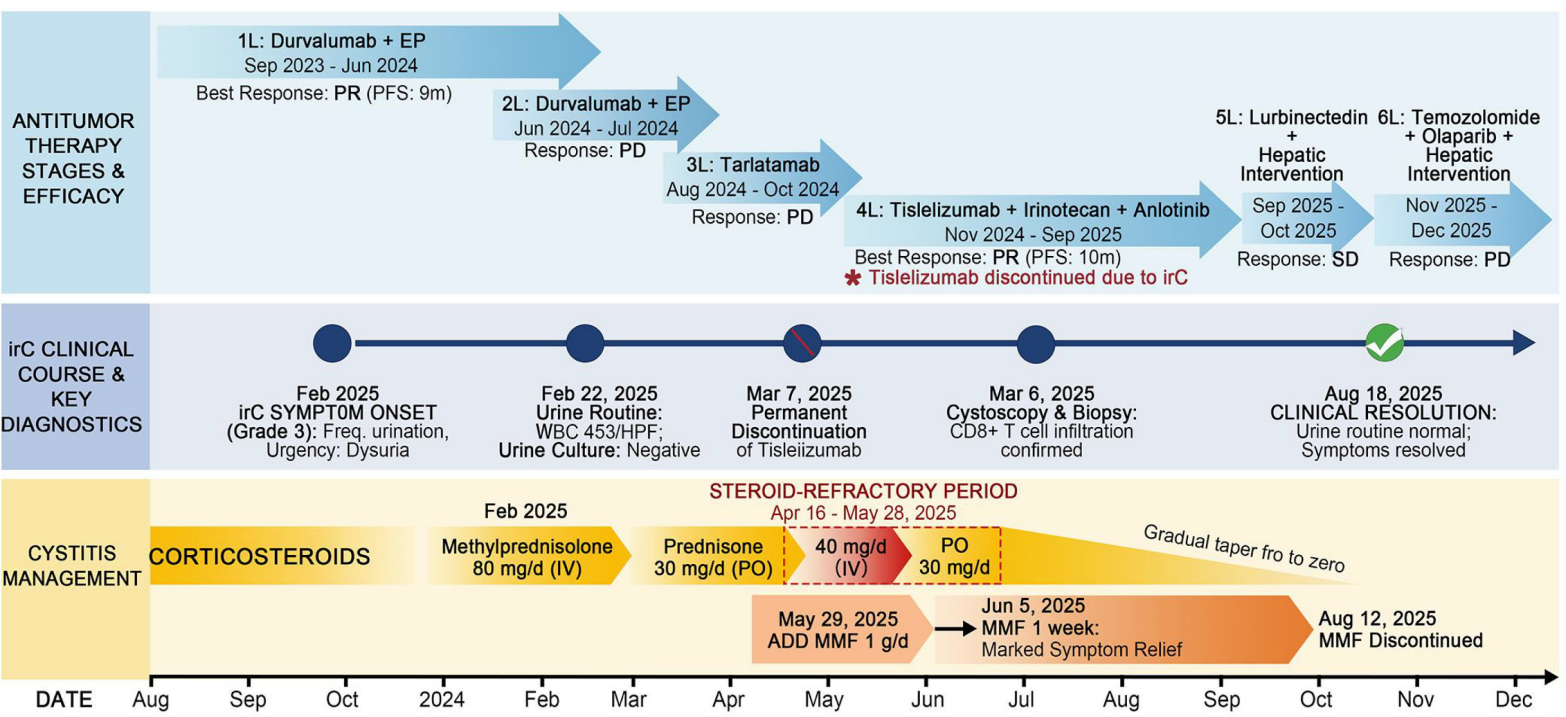
Timeline of the patient’s anti-tumor treatment, cystitis diagnosis and treatment.

On August 12, 2025, MMF was discontinued due to symptom resolution. Prednisone was gradually tapered: 20 mg/day in August, 5 mg/day in September, and completely discontinued in October 2025. As of December 2025, the patient remained free of recurrent urinary irritation symptoms. During fifth-line (lurbinectedin + hepatic interventional therapy) and sixth-line (temozolomide + olaparib + hepatic interventional therapy) treatments, ICIs were not reinitiated, and the primary tumor remained stable. The patient experienced tumor progression in December 2025; anti-tumor treatment was not performed due to poor performance status, and palliative symptomatic treatment was initiated instead. The entire diagnosis and treatment process is shown in **Figure 3**.

## Discussion

A clear temporal correlation existed between the administration of tislelizumab and the onset of cystitis symptoms. The case demonstrated typical histological features of ICI-mediated toxicity, with no similar adverse events reported during previous immunotherapy cycles, and showed a favorable clinical response to targeted immunosuppressive therapy. Furthermore, anlotinib was continuously administered throughout the entire clinical course, and no existing evidence indicates that anlotinib induces or exacerbates cystitis, nor does it increase the risk of urinary tract irAEs when combined with immune checkpoint inhibitors. Therefore, the immune-related cystitis in this patient was highly suspected to be caused by tislelizumab rather than prior immunotherapeutic agents. The core clinical features of ICI-related cystitis include urinary irritative symptoms (79.1% with frequency, 69.8% with hematuria, 53.5% with dysuria), negative urine cultures (81.4%), and pathological findings dominated by CD3^+^ and CD8^+^ T-cell infiltration (8). A unified definition for glucocorticoid-refractory irAEs has not yet been established; however, for ICI-related liver injury, cases are defined as refractory if symptoms fail to improve, progress, or recur when the glucocorticoid dose is tapered to ≤20 mg/day of prednisone (or an equivalent dose) following initial treatment with 1–2 mg/kg/day of methylprednisolone (4).

The patient in this case initially responded to glucocorticoid treatment but experienced recurrence when the dose was reduced to 15 mg/day, and symptoms persisted even after increasing the dose to 40 mg/day, fully meeting the above definition. The underlying mechanisms of refractoriness may be related to the following factors. First, pathological examination revealed bladder mucosa infiltration dominated by CD8^+^ T cells, which may overexpress multidrug resistance protein 1 (MDR1). This protein increases glucocorticoid efflux, impairing the drug’s ability to inhibit T cell cytotoxicity (9). Second, bladder epithelial cells constitutively express PD-L2; ICIs block the PD-1/PD-L2 pathway, causing epithelial cells to lose immune tolerance and triggering persistent immune attack. Glucocorticoids are unable to reverse the established local inflammatory microenvironment (10). Third, unlike hepatitis (which is predominantly T cell-mediated), some cases of cystitis may involve antibody-mediated humoral immune injury, which is significantly less responsive to glucocorticoids (11).

MMF, an inhibitor of inosine monophosphate dehydrogenase, selectively blocks guanine nucleotide synthesis in T and B lymphocytes, inhibiting their proliferation and activation. It is recommended as a second-line therapy for glucocorticoid-refractory immune-related adverse events (irAEs), such as pulmonary toxicity, hepatobiliary toxicity, and other manifestations, according to the NCCN and ESMO guidelines. Its efficacy has been validated in multi-system irAEs, such as Liver irAEs, a meta-analysis showed that MMF has an 82.9% response rate in treating glucocorticoid-refractory ICI-related hepatitis, with a median onset time of 36 days; 81.8% of patients achieved recovery of liver function to CTCAE Grade ≤1 (6). Kidney irAEs, in ICI-related acute interstitial nephritis, MMF can reduce serum creatinine in glucocorticoid-refractory patients within 3–4 days, avoiding the need for dialysis (12). For cholestatic irAEs, MMF combined with ursodeoxycholic acid can improve ALP and GGT levels, enabling safe glucocorticoid dose reduction (13).

In this case report, MMF was selected as a second-line therapy for glucocorticoid−refractory ICI-related cystitis following comprehensive multidisciplinary team (MDT) evaluation, taking into account pathological mechanism, pharmacological properties, and the patient’s long-term oncologic safety. Notably, high-dose glucocorticoid pulse therapy was not adopted because the patient exhibited obvious glucocorticoid dependence and refractoriness with rapid relapse during tapering. Previously, only one case of glucocorticoid-refractory ICI-related cystitis was reported, which was successfully treated with infliximab (5 mg/kg) (5). However, infliximab carries risks of increased infections (e.g., listeriosis, aspergillosis) and induction of autoimmune diseases (14). Given the CD8^+^ T-cell-mediated immune injury in this patient, MMF can specifically target activated CD8^+^ T cells and suppress their proliferation and cytotoxicity, which is highly consistent with the underlying pathogenesis. In contrast, after MMF treatment in this case, symptoms relieved within 1 week, and indicators returned to normal within 8 weeks, significantly faster than the median onset time of MMF in hepatitis (36 days). This may be attributed to the bladder being a local organ, allowing easy drug access to the inflammatory site. Only mild diarrhea occurred, with no severe adverse events such as infection or myelosuppression, showing lower infection risk than infliximab. And smooth glucocorticoid tapering, prednisone was gradually reduced from 30 mg/day to complete discontinuation, resolving the core issue of “glucocorticoid dependence-refractoriness” and aligning with the “steroid-sparing” advantage of MMF (15).

A recent single-center cohort study reported the first application of tofacitinib, a JAK inhibitor, in the treatment of ICI-related ureteritis and cystitis. Two patients with steroid tapering failure achieved significant clinical remission and successful steroid tapering after treatment with low-dose corticosteroids combined with tofacitinib (5 mg twice daily), confirming that JAK inhibitors serve as an effective second-line option for corticosteroid-refractory urinary tract irAEs (16). By blocking the JAK-STAT signaling pathway to modulate systemic immune activation, this class of agents is more suitable for lesions involving the entire urothelium of both the ureter and bladder. In our case, combination therapy with MMF and corticosteroids also yielded remarkable clinical remission, with only transient mild diarrhea as an adverse event and no severe complications such as infection or myelosuppression observed. Collectively, JAK inhibitors and MMF provide diverse second-line therapeutic options for corticosteroid-refractory ICI-associated urinary tract irAEs, offering important references for the clinical management of this rare type of irAEs.

The potential mechanism underlying the efficacy of MMF in treating immune-related cystitis is likely consistent with that reported in conditions such as immune-related hepatitis, all rooted in a conserved core mode of action for steroid-refractory irAEs (6, 12, 15). Upon *in vivo* conversion to mycophenolic acid, MMF selectively inhibits inosine monophosphate dehydrogenase (IMPDH), the rate-limiting enzyme in *de novo* purine synthesis. Lymphocytes (both T and B cells) are entirely dependent on this pathway for proliferation, whereas somatic cells can compensate via the purine salvage pathway, endowing MMF with the ability to specifically target activated immune cells (6, 15). In the present case, pathological examination confirmed a predominance of CD8^+^ T cell infiltration in the bladder lamina propria; these cells mediate urothelial injury through cytotoxicity and the secretion of proinflammatory cytokines, a mechanism analogous to the pathogenic role of CD8^+^ T cell infiltration in steroid-refractory ICI-induced hepatitis. Furthermore, MMF directly addresses the key mechanisms underlying steroid resistance commonly observed in irAEs: pathogenic CD8^+^ T cells may overexpress multidrug resistance protein 1 (MDR1), which promotes glucocorticoid efflux (9), and glucocorticoids are ineffective at eliminating pathogenic T cells that have already undergone clonal expansion (4). Notably, the predominant infiltration of CD8^+^ T cells in this case further underscores the therapeutic advantage of MMF over glucocorticoids: glucocorticoids preferentially suppress CD4^+^ T cells but exert limited effects on terminally differentiated CD8^+^ cytotoxic T cells (11), whereas MMF inhibits the proliferation of all T cell subsets and reduces the secretion of cytotoxic molecules (e.g., perforin, granzyme B) and proinflammatory cytokines (e.g., IFN-γ, TNF-α) by CD8^+^ T cells (8, 15), ultimately leading to rapid symptom relief and normalization of urinary inflammatory markers.

Whether to reinitiate ICIs after controlling ICI-related adverse events is a complex clinical decision, especially for tumor types with limited treatment options (e.g., small-cell lung cancer) where ICIs may provide long-term benefits. A meta-analysis showed that the recurrence rate of irAEs after ICI reinitiation is approximately 22% (95% CI: 15%–30%), with grade 3–4 recurrence rate of about 8%, and most recurrences occur 4–8 weeks after reinitiation (6). Literature shows that reinitiating ICIs is associated with a significantly increased risk of recurrence (7 out of 9 recurrent cases were attributed to reinitiation), but 97.7% of patients can achieve symptom improvement through standardized treatment, providing the possibility of reinitiation for patients where benefits outweigh risks (8).

For patients who responded to ICI treatment (e.g., PFS of 10 months in the fourth-line treatment of this case), reinitiating ICIs may provide sustained anti-tumor benefits. For glucocorticoid-refractory irAEs, the risk of irAE recurrence after reinitiation may be higher. However, considering that ICI-related cystitis is generally controllable and not life-threatening, if disease progression occurs again, we may consider reinitiating ICIs after weighing the need for tumor control against the risk of organ injury.

## Conclusion

This case is the first to confirm that MMF can effectively treat Tislelizumab-related glucocorticoid-refractory immune-related cystitis. It has the advantages of fast onset and good safety, providing a new treatment option for this rare adverse event. Additionally, this case fully presents the multi-line treatment process, analyzes the basis and risks of ICI reinitiation based on guidelines and literature, and proposes the decision-making principle of “prioritizing alternative treatment evaluation and carefully balancing benefits and risks,” providing a reference for clinical management of similar cases. Future prospective studies are needed to validate the efficacy and safety of MMF in ICI-related cystitis, while exploring biomarkers for ICI reinitiation to optimize individualized treatment regimens.

## Data Availability

The raw data supporting the conclusions of this article will be made available by the authors, without undue reservation.
